# *In vitro* Evaluation of the Safety of Adalimumab for the Eye Under HTLV-1 Infection Status: A Preliminary Study

**DOI:** 10.3389/fmicb.2020.522579

**Published:** 2020-12-23

**Authors:** Hisako Kurozumi-Karube, Koju Kamoi, Naoko Ando, Minami Uchida, Isao Hamaguchi, Kyoko Ohno-Matsui

**Affiliations:** ^1^Department of Ophthalmology & Visual Science, Graduate School of Medical and Dental Sciences, Tokyo Medical and Dental University (TMDU), Tokyo, Japan; ^2^Department of Safety Research on Blood and Biological Products, National Institute of Infectious Diseases, Tokyo, Japan

**Keywords:** human T-cell leukemia virus type 1, anti-TNF-α antibody, adalimumab, safety assessment, side effect, HTLV-1 uveitis, ocular inflammation, ocular infiltration

## Abstract

Adalimumab (ADA), a fully human monoclonal tumor necrosis factor (TNF)-α antibody, is one of the most widely used biologics in the treatment of inflammatory diseases. However, ADA can exacerbate infectious conditions, induce paradoxical reactions such as inflammation, and cause neoplasia. Human T-cell leukemia virus type 1 (HTLV-1) is an infectious agent that induces inflammation and neoplastic infiltration in the eye. To date, numerous HTLV-1 carriers have been treated with adalimumab to suppress inflammation out of necessity, when standard anti-inflammatory drugs such as steroids and immunosuppressive agents have proven inadequate to control the inflammation. Here, we clarify the safety of adalimumab for the eye under HTLV-1 infectious conditions *in vitro*. We used the adult retinal pigment epithelial cell line (ARPE)-19 cell line as ocular resident cells, and used MT2 and TL-Om1 as HTLV-1-infected cells. ARPE-19 and MT2/TL-Om1 were co-cultured, and then adalimumab was administered. Production of cytokines and chemokines, TNF-α receptor (TNF-R), HTLV-1 proviral load (PVL), and apoptosis were measured to assess the effects of adalimumab. Contact between ARPE-19 and MT2/TL-Om1 produced inflammatory cytokines such as TNF, interleukin (IL)-6, IL-8 and IL-10, and transduced chemokines such as interferon-inducible protein-10 (IP-10), monocyte chemotactic protein-1 (MCP-1), monokine induced by interferon-γ (MIG), and regulated on activation, normal T cell expressed and secreted (RANTES). No inflammatory cytokines and chemokines were exacerbated by adalimumab. Expression of TNF-R on ARPE-19 and MT2/TL-Om1 cells, HTLV-1 PVLs of MT2/TL-Om1 cells, and cell growth rate and apoptotic rate of ARPE-19 were unaffected by adalimumab. In conclusion, adalimumab does not appear to exacerbate HTLV-1-associated inflammatory conditions in the eye or increase PVL in HTLV-1-infected T cells. These data suggest that adalimumab could be used safely for the eye under HTLV-1 infectious conditions from the perspective of *in vitro* assessment.

## Introduction

Tumor necrosis factor (TNF)-α antibody was introduced in the late 1990s as a molecularly targeted agent and has since seen wide use against a variety of inflammatory diseases, including rheumatoid arthritis (RA; Bathon et al., 2000), psoriasis (Leonardi et al., 2003), ankylosing spondylitis (Braun et al., 2005), ulcerative colitis (Rutgeerts et al., 2005), and inflammatory bowel disease (Rutgeerts et al., 2004).

Adalimumab (ADA), a fully human monoclonal TNF-α antibody, is most widely used as a biologic therapy for a variety of inflammatory diseases (Mullard, 2018). However, multiple side effects have been reported from adalimumab, including induction of infections, malignancies, and paradoxical reactions such as exacerbation of inflammation (Scheinfeld, 2005).

Human T-cell leukemia virus type 1 (HTLV-1) is known to be prevalent in the southwestern part of Japan, sub-Saharan Africa, South America, the Caribbean area, and foci in the Middle East and Australo-Melanesia (Gessain and Cassar, 2012). A recent investigation revealed the infection was not a local problem but spread all over the world. An estimated 20 million people carry the virus worldwide (Watanabe, 2011). Infection by HTLV-1 causes various human diseases, representing hematological neoplasms such as adult T-cell leukemia/lymphoma (ATL; Uchiyama et al., 1977), and inflammatory diseases such as HTLV-1-associated myelopathy (HAM; Osame et al., 1986) and HTLV-1 uveitis (HU; Mochizuki et al., 1992; Kamoi, 2020).

In the eye, HTLV-1 shows pathogenicity for inflammation and neoplastic infiltration (Kamoi and Mochizuki, 2012a; Kamoi, 2020). HU is a defined clinical entity caused by HTLV-1 and represents the most common sight-threatening ocular inflammatory disease in HTLV-1 endemic countries (Terada et al., 2017a; Kamoi et al., 2020). Significant amounts of cytokines and chemokines can be detected in intraocular fluid from HU patients. These cytokines and chemokines cause intraocular inflammation and result in irreversible ocular tissue damage (Kamoi and Mochizuki, 2012a,b). Proviral load (PVL) is also reported to be closely related to the development of HTLV-1-associated diseases (Iwanaga et al., 2010) and is associated with HU activity (Ono et al., 1998).

Another important HTLV-1-related disorder in the eye is ATL-related ocular manifestations (Liu et al., 2010). Our recent nationwide survey identified ocular infiltration as the most frequent manifestation among ATL patients (Kamoi et al., 2018). ATL might also sometimes originate in the eye (Maruyama et al., 2013). A number of studies have identified neoplastic transformation from HTLV-1-infected T cells to ATL cells as clearly related to increased PVL (Okayama et al., 2004; Iwanaga et al., 2010).

To date, numerous HTLV-1 carriers have been treated with adalimumab to suppress inflammation out of necessity, when standard anti-inflammatory drugs such as steroids and immunosuppressive agents have proven inadequate to control the inflammation (Umekita et al., 2015; Umekita and Okayama, 2020). However, information is lacking about the safety of adalimumab for the eye under conditions of HTLV-1 infection. If the use of adalimumab in HTLV-1 carriers affects immunity, HTLV-1-associated disease in the eye might arise. In other words, there is a possibility that adalimumab could instead induce HTLV-1-associated intraocular inflammation (i.e., HU) and transformation of HTLV-1-infected T cells to neoplastic ATL cells in the eye. Looking back to clinical practice, the guidelines for the use of adalimumab make no mention of screening for HTLV-1 infection before starting treatment.

Recently, we reported an assessment of infliximab for the eye under HTLV-1 infection status *in vitro* (Uchida et al., 2019). In Japan, infliximab was the first biologic introduced into the field of ophthalmology, but has only been approved for use against a single pathology, Behçet disease (Ohno et al., 2019). On the other hand, adalimumab can be used for non-infectious uveitis such as sarcoidosis, Vogt-Koyanagi Harada disease, and many other diseases (Jaffe et al., 2016). In other words, the number of patients indicated for the application of adalimumab is much greater than the number of patients indicated for the application of infliximab. Information on adalimumab is thus much more valuable for ophthalmologists, who previously did not need to check for HTLV-1 infection before administering adalimumab. In addition, adalimumab is a fully human monoclonal TNF-α antibody, whereas infliximab is a chimeric monoclonal TNF-α antibody. These structural differences might result in different effects on the eye under conditions of HTLV-1 infection.

The present *in vitro* study therefore investigated the possible effects of adalimumab on the eye under HTLV-1 infection status using an ocular cell line and HTLV-1-infected T-cell lines. A retinal pigment epithelium (RPE) cell line was chosen as the ocular cell line, because the RPE plays a major role in the blood-ocular barrier through which HTLV-1-infected cells invade into the eye (Kamoi and Ohno-Matsui, 2019), as well as a role in the maintenance of immunological homeostasis in the eye (Holtkamp et al., 2001; Mochizuki et al., 2013).

## Materials and Methods

### Cell Culture

As ocular cells, the adult retinal pigment epithelial cell line (ARPE)-19 human retinal pigment epithelial cell line (American Type Culture Collection, Manassas, VA), a spontaneously immortalized cell line, was cultured in Dulbecco’s modified Eagle’s medium (Wako Pure Chemical Corporation, Osaka, Japan) supplemented with 10% heat-inactivated fetal bovine serum (FBS; GE Healthcare Japan, Tokyo, Japan) and 1% penicillin and streptomycin (P/S) antibiotic solution. The MT2 cell line and TL-Om1 were used as HTLV-1-infected T-cell lines, and Jurkat cells were used as a control T-cell line. MT2, TL-Om1 and Jurkat cells were cultured in RPMI 1640 medium (Wako Pure Chemical Corporation) with the same supplements. All cell lines were incubated at 37°C, under 5% CO_2_. Cell culture inserts with 0.4-μm pores (Greiner Bio-One, Kremsmünster, Austria) were used to prevent the contamination of ARPE cells by other cells.

### TNF-α Inhibitor

Adalimumab (Humira®; AbbVie, Chicago, IL) was used as an anti-TNF-α antibody. In line with the previous established method (Fukui et al., 2017), adalimumab concentrations of 0.1, 1.0, and 10 μg/ml were used.

### Cytometric Bead Array

We seeded 1.5 × 10^5^ ARPE cells in 6-well cell culture plates and incubated them for 24 h. Subsequently, 5 × 10^5^ MT2, TL-Om1 or Jurkat cells were co-cultured following 48 h with 0, 0.1, 1.0, or 10 μg/ml of adalimumab. We performed cytometric bead array (CBA) using culture supernatants and CBA human inflammation cytokine kits (BD Bioscience, San Jose, CA). FCAP Array version 3.0 software (BD Bioscience) was used for analyses in accordance with the instructions from the manufacturer. Cytokines measured by the kits included interleukin (IL)-6, IL-1β, IL-12p70, IL-8, IL-10, and TNF, and chemokines included regulated on activation, normal T cell expressed and secreted [RANTES; also known as C-C motif chemokine ligand (CCL)5], monokine induced by interferon-γ [MIG; also known as C-X-C motif chemokine ligand (CXCL)9], monocyte chemotactic protein-1 (MCP-1; also known as CCL2), and interferon-inducible protein-10 (IP-10; also known as CXCL10). Minimum limits of detection for cytokines/chemokines were as follows: IL-6, 2.5 pg/ml; IL-1β, 7.2 pg/ml; IL-12p70, 1.9 pg/ml; IL-8, 0.2 pg/ml; IL-10, 3.3 pg/ml; TNF, 3.7 pg/ml; RANTES, 1.0 pg/ml; MIG, 2.5 pg/ml; MCP-1, 2.7 pg/ml; and IP-10, 2.8 pg/ml.

### Cell Growth Analysis

ARPE-19 cells (2 × 10^4^) were co-cultured with three times the number of MT2, TL-Om1 or Jurkat cells using 0.4-μm pore insert, with 0 or 10 μg/ml of adalimumab in 24-well cell culture plates. After 0, 24, 48, or 72 h of co-culture, we removed the supernatants, trypsinized ARPE-19, and counted the number of ARPE-19 cells under light microscopy.

### TNF-α Receptor Analysis

ARPE-19 cells (1.5 × 10^5^) were co-cultured with three times the number of MT2, TL-Om1, or Jurkat cells using a 0.4-μm pore insert, with 0 or 10 μg/ml of adalimumab. MT2 or TL-Om1 cells were cultured in RPMI1640 containing 10% FBS and 1% P/S with 0 or 10 μg/ml of adalimumab. Analysis of surface expression of TNF-R1 and TNF-R2 on ARPE-19, MT2 and TL-Om1 were performed using human TNF RI/TNFRSF1A PE-conjugated antibody (R&D Systems, Minneapolis, MN) and human TNF RII/TNFRSF1B fluorescein-conjugated antibody (R&D Systems) compared to an isotype control antibody. Samples were measured using FACSCalibur (BD Biosciences) and analyzed with CellQuest software (BD Biosciences).

### Immunofluorescence Microscopy

ARPE-19 cells were cultured on glass plates in a cell culture plate (AGC Techno Glass, Shizuoka, Japan) for 24 h, then co-cultured with MT2, TL-Om1 cells using cell culture inserts (Thermo Fisher Scientific, San Jose, CA) for 48 h. ARPE-19 cells were permeabilized and fixed by cold fixing buffer (methanol/acetone, 1:1) at −20°C for 20 min. After blocking with 5% normal goat serum in phosphate-buffered saline for 15 min, cells were incubated in the diluted primary antibodies for 1 h at room temperature, followed by incubation with Alexa Fluor 488-labeled anti-rabbit secondary antibody (Abcam, Cambridge, MA) for 1 h at room temperature. After nuclear staining with 4',6-diamidino-2-phenylindole, cells were scanned using a TCS-SP8 microscope (Leica Micro Systems, Wetzlar, Germany). TNF-R1 polyclonal antibody (Bioss Antibodies, Woburn, MA) and TNF-R2 polyclonal antibody (Proteintech, Chicago, IL), or rabbit IgG control (Abcam) were used as primary antibodies.

### Measurement of HTLV-1 Proviral Load

MT2 or TL-Om1 cells were cultured in RPMI1640 containing 10% FBS and 1% P/S with 0 or 10 μg/ml of adalimumab. EZ1 Virus Mini Kits v2.0 (Qiagen, Hilden, Germany) was used to prepare the DNA from each sample. PVL of HTLV-1 in cells was measured using quantitative real-time PCR assay as described previously (Matsuda et al., 2005; Fukui et al., 2017; Uchida et al., 2019). PVL was quantified using the HTLV-1 Tax primer (forward, 5'-CCCACTTCC CAGGGTTTGGA-3'; reverse, 5'-GGCCAGTAGGGCG TGA-3') and probe (5'-FAM-CCAGTCTACGTGTTTGGA GACTGTGTACA-TAMRA-3'). Glyceraldehyde-3-phosphate dehydrogenase was used as the internal control.

### Annexin V Staining

Evaluations of apoptosis and cell death were performed using Annexin V-FITC/PI double-staining assay kits (MBL, Nagoya, Japan) in line with the instructions from the manufacturer. Briefly, cells were stained using propidium iodide (PI) and fluorescein isothiocyanate (FITC)-conjugated annexin V for 15 min in room air. Fluorescence-activated cell sorting (FACS) analysis was performed immediately using a FACSCalibur flow cytometer and CellQuest software. PI-negative, annexin V-positive cells were defined as apoptotic. ARPE-19 cells were used at 2 × 10^4^ cells, and MT2, TL-Om1, or Jurkat cells were used at 6 × 10^4^ cells, respectively, with cell culture inserts.

### Statistical Analysis

After one-way analysis of variance, Student’s unpaired *t*-test or Welch’s unpaired-*t*-test after Bonferroni correction for multiple testing was used to determine significant differences in levels of cytokines, numbers of ARPE-19 cells and percentages of apoptotic cells. Values of *p* < 0.05 were considered significant. All statistical analyses were performed using EZR (Saitama Medical Center, Jichi Medical University, Saitama, Japan), a graphical user interface for R (The R Foundation for Statistical Computing, Vienna, Austria). More precisely, EZR is a modified version of R Commander designed to add statistical functions frequently used in biostatistics.

## Results

### Production of Inflammatory Cytokine and Chemokines

**Figure 1:**
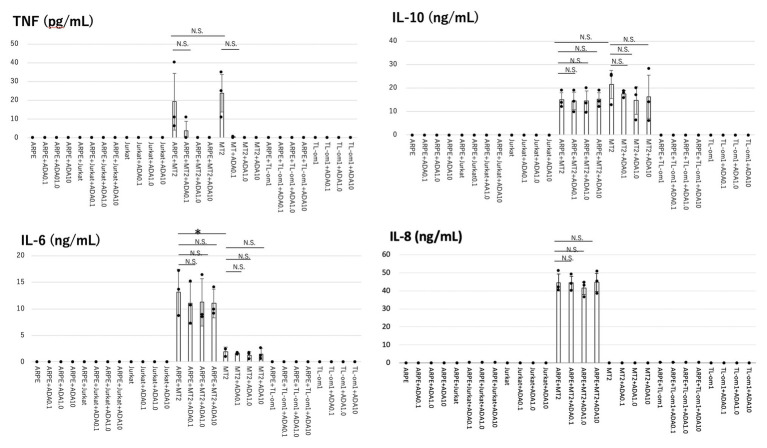
Levels of inflammatory cytokines measured in the culture supernatants of adult retinal pigment epithelial cell line (ARPE)-19, MT2, TL-Om1, or Jurkat, and ARPE-19 co-cultured with MT2, TL-Om1, or Jurkat, with addition of adalimumab (ADA). ARPE cells (1.5 × 10^5^ cells) were co-cultured with 5 × 10^5^ MT2, TL-Om1 or Jurkat cells following 48 h with 0, 0.1, 1.0, or 10 μg/ml of ADA. Production levels of IL-6, IL-10, and IL-8 did not change with adalimumab addition. Adalimumab decreased tumor necrosis factor (TNF) secretion in a concentration-dependent manner. TNF was not detected with ≥1.0 μg/ml of adalimumab. Data were obtained from three independent experiments. Error bars represent SD (^*^p < 0.05; NS, not significant). ADA, adalimumab.

Levels of cytokines (IL-6, IL-8, IL-1β, IL-12p70, IL-10, and TNF) and chemokines (RANTES, MIG, MCP-1, and IP-10) secreted by each of ARPE-19, MT2, Jurkat, ARPE-19 co-cultured with MT2/TL-Om1, and ARPE-19 co-cultured with Jurkat were measured (Figure 1).

In terms of cytokines, MT2 spontaneously secreted IL-6, IL-10, and TNF. IL-8 was not secreted from MT2, whereas TL-Om1 did not spontaneously secrete any cytokines. ARPE-19 did not secrete any cytokines measured in this experiment.

IL-6, IL-10, and TNF were produced in ARPE-19 co-cultured with MT2, but not in ARPE-19 alone or in co-culture with TL-Om1 and Jurkat. IL-8 was induced only through contact between ARPE-19 and MT2 cells. Compared to MT2 alone, levels of IL-6 and IL-8 were increased. On the other hand, levels of TNF and IL-10 appeared similar in ARPE-19 co-cultured with MT2. No cytokine release was detected by contact between ARPE-19 and Tl-Om1. Levels of IL-12p70 and IL-1β were below the limits of detection under all conditions upon co-culture with ARPE-19 cells (data not shown).

**Figure 2:**
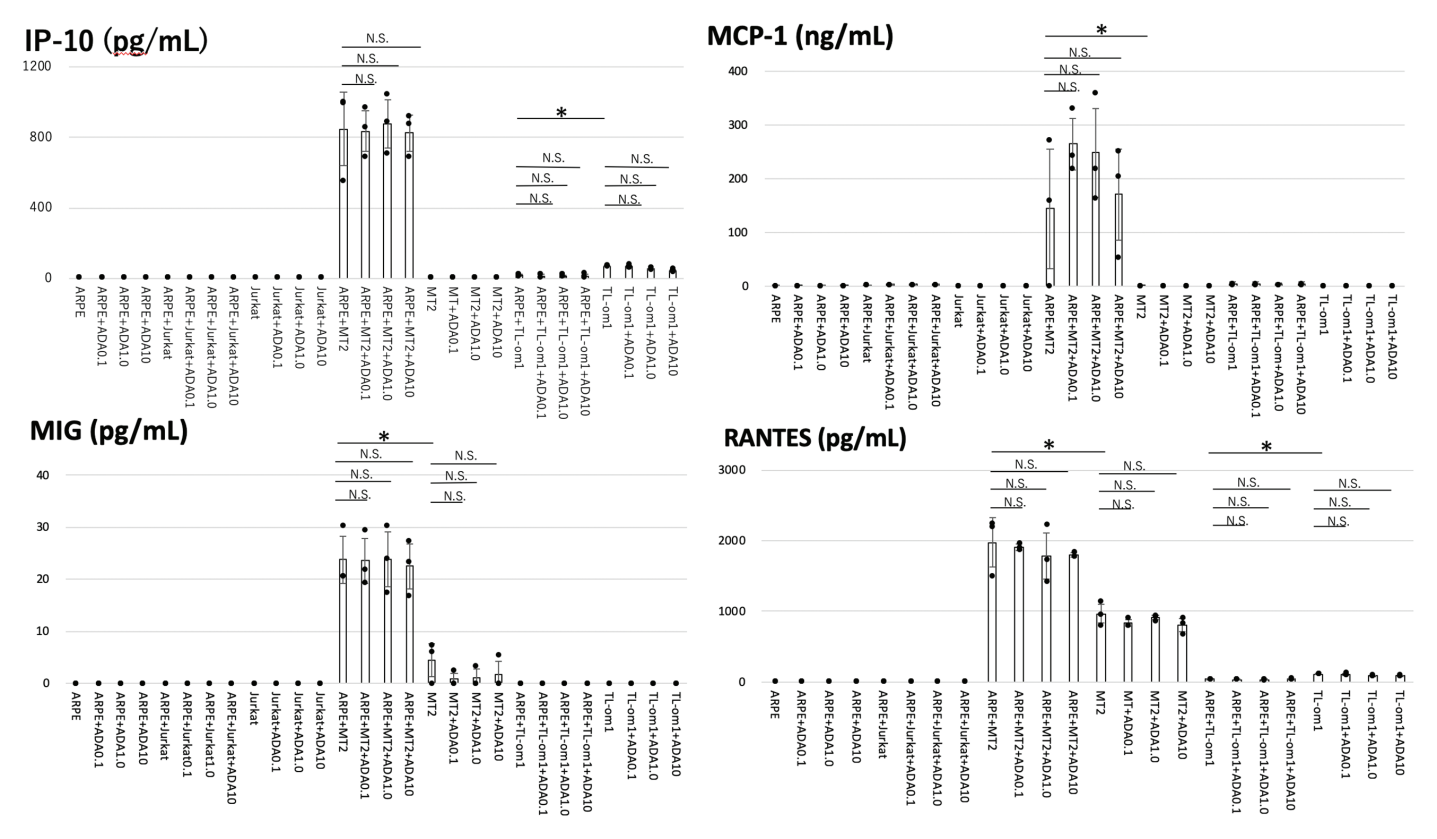
Levels of chemokines were measured in the culture supernatants of ARPE-19, MT2, TL-Om1, or Jurkat, and ARPE-19 co-cultured with MT2, TL-Om1 or Jurkat with addition of adalimumab. Coculture conditions were the same as in Figure 1. Adalimumab concentrations were 0.1, 1.0, or 10 μg/ml. Production levels of interferon-inducible protein-10 (IP-10), monocyte chemotactic protein-1 (MCP-1), monokine induced by interferon-γ (MIG), and regulated on activation, normal T cell expressed and secreted (RANTES) in co-culture of ARPE-19 cells and MT2 or TL-Om1 cells did not change with addition of adalimumab. Error bars represent SD (^*^p < 0.05; NS, not significant). ADA, adalimumab.

As for chemokines, MT2 spontaneously secreted MIG and RANTES. IP-10 and MCP-1 were not secreted from MT2. TL-Om1 spontaneously secreted RANTES and IP-10, but did not secrete MIG or MCP-1. ARPE-19 did not secrete any chemokines measured in this experiment (Figure 2).

All measured chemokines (RANTES, MIG, MCP-1, and IP-10) were produced in significantly greater quantities by ARPE-19 co-cultured with MT2 than by ARPE-19 co-cultured with Jurkat. IP-10 and MCP-1 were induced through the contact. Compared to MT2 alone, levels of all chemokines were also increased. As for production of chemokines by ARPE-19 co-cultured with TL-Om1, RANTES and IP-10 were decreased in comparison with production by TL-Om1 alone.

### Changes in Cytokine and Chemokine of ARPE-19 Co-cultured With MT2 or TL-Om1 Cells Treated With Adalimumab

Cytokine and chemokine levels in culture supernatant of ARPE-19 co-cultured with MT2, TL-Om1, and Jurkat cells as well as ARPE-19 alone were measured at 48 h after addition of adalimumab.

Among the inflammatory cytokines, production levels of IL-6, IL-8, and IL-10 were unaltered by addition of adalimumab at each concentration in all co-culture combinations. In ARPE-19 co-cultured with MT2, TNF tended to be inhibited in a concentration-dependent manner, but did not show any significant difference at 0.1 μg/ml of adalimumab. TNF was completely inhibited at concentrations of adalimumab ≥1.0 μg/ml (Figure 1). A similar effect was observed with MT2 alone.

Of chemokines, production levels of RANTES, MIG, MCP-1, and IP-10 were unaltered by addition of adalimumab at each concentration in ARPE-19 co-cultured with MT2 or TL-Om1 (Figure 2).

### Changes in Cell Growth in ARPE-19 Treated With Adalimumab

**Figure 3:**
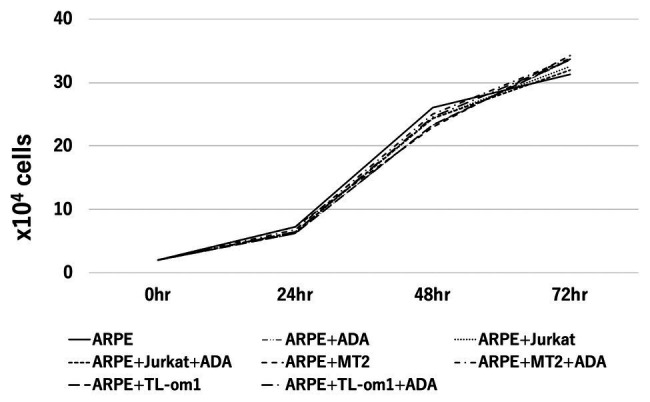
Cell growth curve of ARPE-19, showing number of ARPE-19 cells per well at 0, 24, 48, and 96 h. The number of ARPE cells was 2.0 × 10^4^ at the beginning of culture. No significant differences among numbers of ARPE-19 cells co-cultured with MT2 or TL-Om1 cells with or without 10 μg/ml of adalimumab were seen at each point. The number of cells was counted three times independently. ADA, adalimumab.

Cell growth rates of ARPE-19 co-cultured with MT2 or TL-Om1 and treated with adalimumab were examined (Figure 3). As controls, cell growth rates were measured for ARPE-19 alone, ARPE-19 treated with adalimumab, ARPE-19 co-cultured with Jurkat, and ARPE-19 co-cultured with Jurkat treated with adalimumab. Numbers of ARPE-19 cells were counted after 0, 24, 48, and 72 h of co-culturing. Growth curves showed a time-dependent form and were similar among ARPE-19, ARPE-19 co-cultured with MT2, TL-Om1, or Jurkat, and ARPE-19 co-cultured with MT2, TL-Om1, or Jurkat treated with adalimumab. Addition of adalimumab under this co-cultured condition did not affect the growth rate of ARPE-19 cells (Figure 3).

### Expressions of TNF-R1 and TNF-R2 on ARPE-19 Cells Co-cultured With MT2 or TL-Om1 Cells Treated With Adalimumab

**Figure 4:**
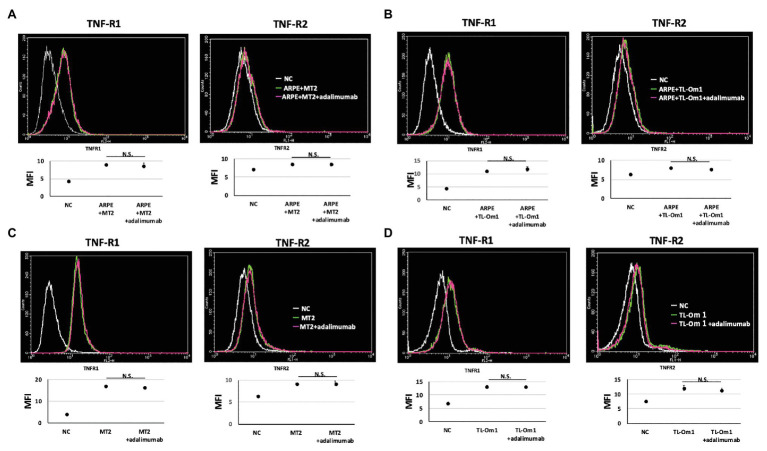
TNF-R1 and TNF-R2 on ARPE-19 in fluorescence-activated cell sorting (FACS) analysis. Results are representative of three experiments and show that expressions of TNF-R1 and -R2 on ARPE-19 cells (1.5 × 10^5^ cells) co-cultured with MT2 cells **(A)** or TL-Om1 cells (5 × 10^5^ cells; **B**) were unaffected by 10 μg/ml of adalimumab. Expressions of TNF-R1 and TNF-R2 on MT2 cells **(C)** and TL-Om1 cells **(D)** were also unaffected by 10 μg/ml of adalimumab. Histograms (upper figure) and mean fluorescence intensity (MFI; lower figure) for expression of TNF-R1 and TNF-R2. Error bars represent SD. NS, not significant.

**Figure 5:**
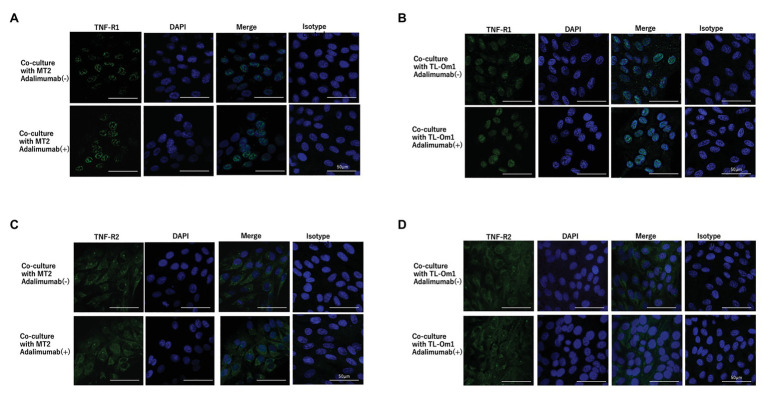
Immunofluorescence staining of TNF-R1 **(A,B)** and TNF-R2 **(C,D)** on ARPE-19 co-cultured with MT2 or TL-Om1. ARPE-19 cells were treated with rabbit monoclonal anti-TNF-R1 or anti-TNF-R2 antibodies followed by Alexa Fluor 488-labeled anti-rabbit secondary antibody. DAPI was used to stain the nucleus. No significant changes in expressions of TNF-R1 or TNF-R2 are seen for ARPE-19 treated with or without 10 μg/ml of adalimumab. **(A,B)** Green color represents TNF-R1 expression and blue represents DAPI. **(C,D)** Green color represents TNF-R2 and blue represents DAPI. Isotype control indicates staining control with rabbit IgG control as the first antibody.

To identify changes in expressions of TNF-R1 and TNF-R2 caused by adalimumab, we assessed expressions of TNF-R1 and -R2 on ARPE-19 and MT2 or TL-Om1 at 48 h after addition of adalimumab, by performing FACS analysis. Treatment with 10 μg/ml of adalimumab resulted in no significant change in mean fluorescence intensity (MFI) of TNF-R1‐ or TNF-R2-labeled ARPE-19 cells (Figures 4A,B). Addition of adalimumab also did not affect expressions of TNF-R1 or -R2 on MT2 or TL-Om1 (Figures 4C,D). We also performed immunofluorescence staining. Expressions of TNF-R1 (Figure 5A) and TNF-R2 (Figure 5B) on ARPE-19 co-cultured with MT2 cells were unaltered by the presence or absence of adalimumab. Expressions of TNF-R1 (Figure 5C) and TNF-R2 (Figure 5D) on ARPE-19 co-cultured with TL-Om1 cells were also unaltered by the presence or absence of adalimumab.

### Detection of HTLV-1 Proviral DNA in HTLV-1-Infected T Cells Treated With Adalimumab

**Figure 6:**
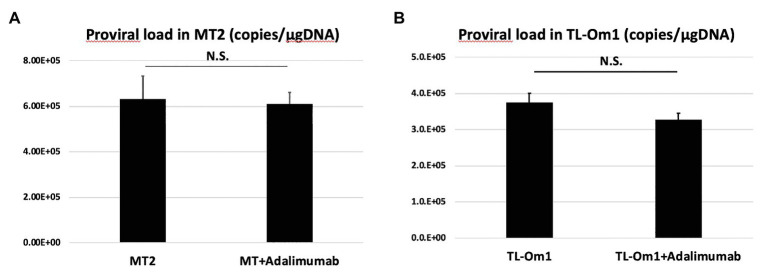
Proviral load (PVL) in MT2 cells **(A)** or TL-Om1 cells **(B)** treated with 10 μg/ml of adalimumab. The number of cells was 5 × 10^5^, respectively. PVL of both cells was unchanged by adalimumab. Error bars represent SD. NS, not significant.

Proviral load in HTLV-1-infected cells is the most frequently used biomarker for prognosis and disease progression (Iwanaga et al., 2010). Therefore, to identify whether adalimumab induces changes in PVL for MT2 or TL-Om1 cells, we measured PVL at 48 h after addition of adalimumab. To assess the potential effects of adalimumab on HTLV-1-infected cells, we checked the PVL of MT2 or TL-Om1 cells at 48 h after addition of adalimumab. Mean PVLs in MT2 and TL-Om1 were 6.32 × 10^5^ and 3.70 × 10^5^ copies/μg DNA, respectively. On the other hand, mean PVLs in MT2 and TL-Om1 treated with adalimumab were 6.02 × 10^5^ and 3.30 × 10^5^ copies/μg DNA, respectively. No significant difference was detected according to the presence or absence of adalimumab (Figure 6).

### Assessment of Apoptosis in ARPE-19 Cells Treated With Adalimumab

**Figure 7:**
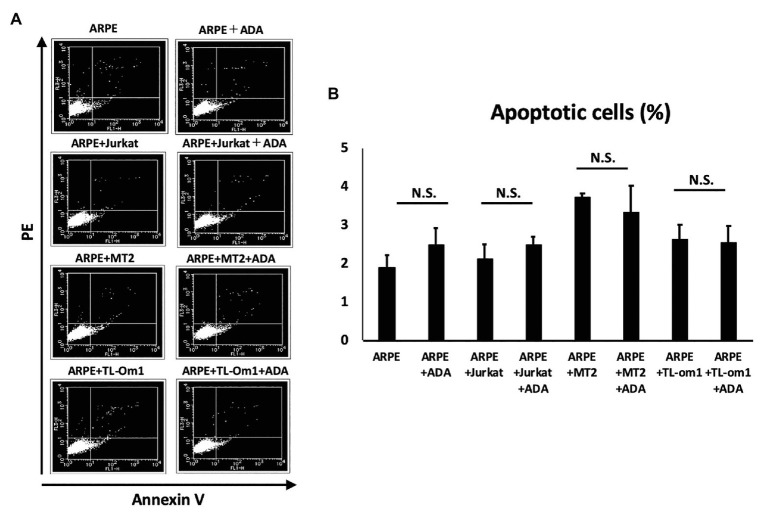
**(A)** Annexin-V staining assay results for apoptosis of ARPE-19 cells. Extent of apoptosis was determined as the percentage of Annexin V-positive, PI-negative cells. **(B)** Rate of apoptotic ARPE-19 cells (2 × 10^4^ cells) in co-culture with MT2, TL-Om1, or Jurkat cells (6 × 10^4^ cells), and MT2, TL-Om1, or Jurkat cells (6 × 10^4^ cells) treated with 10 μg/ml of adalimumab. No significant change in apoptotic rate of ARPE-19 is seen with adalimumab. Error bars represent SD. NS, not significant; ADA, adalimumab.

To assess apoptosis in ARPE-19 cells, we performed annexin V staining of ARPE-19 at 48 h after addition of adalimumab. Apoptosis was seen in 3.74% of ARPE-19 cells co-cultured with MT2 cells, and in 2.63% of ARPE-19 cells co-cultured with TL-Om1 cells. Frequencies of apoptosis for ARPE-19 in each co-culture were 3.34 and 2.55% with addition of adalimumab, respectively. No significant change in apoptotic rate was seen for ARPE-19 cells co-cultured with MT2 or TL-Om1, with or without addition of adalimumab (Figure 7).

## Discussion

This study focused on adalimumab, as one of the most widely used anti-TNF-α antibodies in the treatment of inflammatory diseases (Mullard, 2018), and investigated the effects of adalimumab on the eye under HTLV-1 infection status *in vitro*. The study demonstrated that adalimumab did not cause significant changes in the production of inflammatory cytokines IL-6, IL-8, and IL-10 or chemokines MCP-10, MIG, RANTES, and IP-10, proliferation of ARPE-19 cells, expression of TNF-R1 or -R2, or apoptosis, compared to non-treated ARPE-19 cells under HTLV-1 infectious conditions. In addition, HTLV-1-infected T cells were unaffected by adalimumab in terms of cytokine/chemokine production, TNF-R1 or -R2 expression, and PVL increases. This implies that adalimumab did not exacerbate HTLV-1-related inflammation in the eye, at least from the perspective of *in vitro* experiments.

Globally, HTLV-1 infection has gained attention since the high prevalence among Aborigines in central Australia became widely recognized (Martin et al., 2018). Japan is the most endemic country among the developed nations in terms of HTLV-1 infection, with an estimated 1.08 million infected individuals. Japan is one of the countries where patients with inflammatory diseases are treated with biologic agents. Among HTLV-1-infected patients, many patients need biologics because of severe inflammatory diseases such as RA, psoriasis, or ankylosing spondylitis uncontrollable by standard steroid or immunosuppressive drugs (Umekita et al., 2015). However, little is known about whether biologics can be safely used by infected individuals, particularly in the field of ophthalmology. Biologics may upregulate HTLV-1 activities and change the immunological homeostasis of the eye, which might result in induction of HTLV-1-associated ocular diseases. In fact, we have already reported a case in which an RA patient with HTLV-1 infection received biologics, which resulted in activation of HTLV-1 status and induced HU in the eye (Terada et al., 2017b).

In general, adalimumab is an effective drug for various inflammatory diseases, and many reports have suggested that adalimumab can decrease the levels of various cytokines in patients of inflammatory diseases (Scheinfeld, 2004). However, paradoxical response, or exacerbation of inflammation, is also well known during anti-TNF-α therapy. In particular, the occurrence of uveitis (ocular inflammation) has been reported as a major paradoxical effect (Wendling and Prati, 2014). In addition, HU can be seen among HTLV-1 carriers, which means activation of HTLV-1 by adalimumab might disturb the immunological homeostasis of the eye. We therefore checked the effects of adalimumab on inflammatory cytokines (IL-6, IL-8, IL-1β, IL-12p70, IL-10, and TNF) and chemokines (MCP-10, MIG, RANTES, and IP-10) under HTLV-1 infectious conditions, i.e., under conditions of MT2/TL-Om1 cells in contact with ARPE-19 cells.

Inflammatory cytokine investigations showed that levels of IL-6 and IL-8 increased when MT2 cells came into contact with ARPE-19 (Figure 1). These inflammatory cytokines can lead to damage to ocular tissues, suggesting that HTLV-1 carriers face a potential threat of inflammation in the eye (Uchida et al., 2019). As for TNF-production, despite low-level detection, no significant change was seen when MT2 cells came into contact with ARPE-19, compared to HTLV-1-infected T cells, but mean TNF levels were slightly decreased with this contact. Previous reports have mentioned that this might be explained by the notion that RPE is thought to play a role in local immunosuppressive ability (Mochizuki et al., 2013; Uchida et al., 2019) and this finding was not thought to rule out an immunosuppressive role of RPE.

As for the effect of adalimumab administration, levels of IL-6, IL-8, and IL-10 production were unaffected by adalimumab treatment, suggesting that adalimumab does not exacerbate inflammation in the eye under HTLV-1 infectious conditions. The level of TNF production after contact between HTLV-1-infected cells and ARPE-19 was low, but decreased in a dose-dependent manner with addition of adalimumab. Complete suppression of TNF was also seen with higher-dose adalimumab administration (Figure 1). These results suggest that adalimumab might be somewhat effective for reducing immune reactions through inhibition of TNF in the eye.

In terms of chemokines, production of MCP-1, MIG, IP-10, and RANTES increased when MT2 came into contact with ARPE-19 cells, and production levels were unaffected by adalimumab treatment (Figure 2). Decreases in RANTES and IP-10 were seen in the contact between TL-Om1 and ARPE-19, which might relate to the local immunosuppressive ability of RPE as mentioned above (Mochizuki et al., 2013; Uchida et al., 2019). Levels of these chemokines were also unaffected by adalimumab treatment (Figure 2). These results also suggest that adalimumab does not accelerate chemokine-mediated attraction of inflammatory cells into the eye among HTLV-1-infected patients (Charo and Ransohoff, 2006).

Adalimumab-mediated inhibition could be due to interference with the binding of TNF to two known receptors. TNF-R1 binds to soluble TNF and TNF-R2 binds to membrane-bound TNF. TNF-R1 and TNF-R2 are both known to be expressed on RPE (Kociok et al., 1998; Shi et al., 2008; Lee et al., 2015), and changes in these receptors are therefore thought to affect blood-ocular barrier function *via* TNF-mediated inflammation. TNF-R1 and TNF-R2 are also expressed on HTLV-1-infected T cells (Harashima et al., 2001). We therefore checked for changes in TNF-R1 and -R2 expression on ARPE-19 and two types of HTLV-1-infected T cells by FACS analysis and immunofluorescence staining. These experiments identified no changes in TNF-R1 or -R2 expression on ARPE-19 or the two types of HTLV-1-infected T cells through FACS analysis (Figure 4) and immunofluorescence (Figure 5). This suggests that adalimumab has no effect on TNF-mediated intraocular alterations.

We next investigated the effects on PVL in two types of HTLV-1-infected T cells through adalimumab administration. The increase in PVL among HTLV-1-infected T cells relates to the development of ATL, HAM, and HU (Ono et al., 1998; Matsuzaki et al., 2001; Kulkarni and Bangham, 2018). PVL in peripheral blood is also a well-known marker of HTLV-1 disease progression (Demontis et al., 2013). Checking PVL in infected T cells is thus important to clarify the risks of inducing HTLV-1-associated diseases. Considering local circumstances in the eye, the risk of inducing HTLV-1-associated inflammation and transformation to malignant cells in the eye through adalimumab administration is a concern.

As for the effects of adalimumab for MT2 or TL-Om1, the PVL of each cell was also unchanged by adding adalimumab, consistent with previous reports (Umekita et al., 2015; Fukui et al., 2017). This indicates that competent transformation of infected T cells was not stimulated by adalimumab (Figure 6).

Increases in RPE cells undergoing apoptosis critically damage the maintenance of immunological homeostasis in the eye (Mochizuki et al., 2013). In this study, adalimumab treatment did not affect the frequency of RPE apoptosis in co-culture with MT2 or TL-Om1, compared to that in co-culture with control Jurkat cells (Figure 7). In addition, if adalimumab promotes cell growth of HTLV-1-infected RPE, RPE proliferation should be observed. However, adalimumab also did not affect the growth curve of ARPE-19 (Figure 3). These results suggest that adalimumab does not promote the growth of ARPE cells under HTLV-1 infectious conditions.

Various limitations to the present study need to be considered when interpreting the results. We selected MT2 and TL-Om1 as HTLV-1-infected T-cell lines in this investigation. Diversity among MT2 cell lines was pointed out in a previous paper (Nomura et al., 2019). We therefore performed additional experiments using another type of HTLV-1-infected T-cell line, TL-Om1. The same results were obtained in terms of the safety of adalimumab under HTLV-1 infectious conditions *in vitro* with these different types of cells. This study did not reflect all aspects of *in vivo* situations and was instead positioned as a preliminary *in vitro* study. In addition, consideration needs to be given to the aspects of the clinical response to adalimumab in the eye and the effects of adalimumab therapy in patients with inflammatory diseases complicated by HTLV-1 infection. However, this *in vitro* study suggested that the use of adalimumab in HTLV-1-infected patients with inflammatory disease may not aggravate inflammation or induce proliferation in the eye, and this agent may thus be safe to apply in patients with inflammation from an ophthalmological perspective.

## Conclusion

We have shown that adalimumab did not affect production of inflammatory cytokines, expression of TNF-R, cell proliferation, or apoptosis in RPE under HTLV-1 infectious conditions *in vitro*. In addition, adalimumab did not affect increases in PVL in HTLV-1-infected T cells. These results suggest that adalimumab did not exacerbate HTLV-1-related inflammation in the eye. Adalimumab may be safe for use in the eye under HTLV-1 infectious conditions, at least from the perspective of this preliminary *in vitro* assessment.

## Data Availability Statement

All datasets generated for this study are included in the article/supplementary material.

## Author Contributions

HK-K performed the experiments and wrote the draft of the manuscript. KK designed the experiments, analyzed the data, and wrote the manuscript. NA and MU performed the experiments. IH and KO-M contributed to the analysis and interpretation of data, and assisted in the preparation of the manuscript. All authors contributed to the article and approved the submitted version.

### Conflict of Interest

The authors declare that the research was conducted in the absence of any commercial or financial relationships that could be construed as a potential conflict of interest.
